# The role of insight, social rank, mindfulness and self‐compassion in depression following first episode psychosis

**DOI:** 10.1002/cpp.2881

**Published:** 2023-07-12

**Authors:** Jamie R. Hardman, John F. M. Gleeson, César González‐Blanch, Mario Alvarez‐Jimenez, Madeleine I. Fraser, Keong Yap

**Affiliations:** ^1^ School of Behavioural and Health Sciences Australian Catholic University Strathfield New South Wales Australia; ^2^ Healthy Brain and Mind Research Centre, School of Behavioural and Health Sciences Australian Catholic University Fitzroy Victoria Australia; ^3^ Centre for Youth Mental Health University of Melbourne Melbourne Victoria Australia; ^4^ Mental Health Centre University Hospital “Marqués de Valdecilla” Santander Spain

**Keywords:** depression, first‐episode psychosis, insight, mindfulness, self‐compassion, social rank

## Abstract

Gaining awareness of psychosis (i.e., insight) is linked to depression, particularly in the post‐acute phase of psychosis. Informed by social rank theory, we examined whether the insight–depression relationship is explained by reduced social rank related to psychosis and whether self‐compassion (including uncompassionate self‐responding [UCS] and compassionate self‐responding [CSR]) and mindfulness buffered the relationship between social rank and depression in individuals with first episode psychosis during the post‐acute phase. Participants were 145 young people (*M*_age_ = 20.81; female = 66) with first episode psychosis approaching discharge from an early psychosis intervention centre. Questionnaires and interviews assessed insight, depressive symptoms, perceived social rank, self‐compassion, mindfulness and illness severity. Results showed that insight was not significantly associated to depression and thus no mediation analysis was conducted. However, lower perceived social rank was related to higher depression, and this relationship was moderated by self‐compassion and, more specifically, UCS. Mindfulness was related to depression but had no moderating effect on social rank and depression. Results supported previous findings that depressive symptoms are common during the post‐acute phase. The role of insight in depression for this sample is unclear and may be less important during the post‐acute phase than previously considered. Supporting social rank theory, the results suggest that low perceived social rank contributes to depression, and reducing UCS may ameliorate this effect. UCS, social rank and possibly mindfulness may be valuable intervention targets for depression intervention and prevention efforts in the recovery of psychosis.

**Key Practitioner Message**

- Gaining insight following early intervention for first‐episode psychosis (FEP) was not associated with increased depression
- In our sample of individuals recovering from FEP, depression was associated with perceived loss of social rank, lower levels of mindfulness, and self‐compassion.
- Uncompassionate self‐responding but not mindfulness moderated the association between perceived social rank and depression.
- Perceived social rank did not influence depression in participants who had low levels of uncompassionate self‐responding
- Future research should examine the effectiveness of self‐compassion interventions on depression following early intervention for FEP.

## 1 INTRODUCTION

### 1.1 The insight paradox

Impaired insight in psychosis is common and encompasses a lack of awareness of the illness, its signs, symptoms, implications, and need for treatment (Amador et al., 1993). Insight generally improves with treatment and remission of the acute phase. However, improved insight is linked to both positive and negative consequences (Lysaker et al., 2018; Misiak et al., 2016). On the one hand, improved insight is linked to greater treatment adherence and reduced risk of relapse and rehospitalisation (Ramu et al., 2019; Sendt et al., 2015). However, gaining awareness of the illness and its implications is also linked to negative outcomes such as depression, hopelessness and suicidality (Murri et al., 2015). These contradictory effects have been coined as the ‘insight paradox’ (Lysaker et al., 2007).

### 1.2 Insight–depression relationship during the post‐acute recovery phase

Research on the insight paradox has predominantly focused on the insight–depression relationship, with a meta‐analysis finding a small but significant positive association between insight and depression across 50 studies in individuals with psychosis (Murri et al., 2015). Of interest is how the insight–depression relationship unfolds during the years following the first episode of psychosis (post‐acute recovery phase). During this phase, the magnitude of the relationship increases, and depression and suicidality are prevalent (Ayesa‐Arriola et al., 2015; Coentre et al., 2017; Murri et al., 2015). Additionally, depression in the post‐acute phase is a stronger predictor for poor long‐term outcomes (e.g. increased relapse, poor social and vocational outcomes and suicidality) compared to depression in the acute phase (Oosthuizen et al., 2006; Phahladira et al., 2021).

Greater insight relates to and predicts depression in people with first episode psychosis, with medium to large effect sizes (Drake et al., 2004; Phahladira et al., 2021). In this population, depression has been shown to fully mediate the association between insight and suicidality (Roux et al., 2018), and greater insight has predicted attempted suicide (Robinson et al., 2009). Approximately 15% of first episode individuals are known to attempt suicide within 3 years of presenting for treatment, and depression severity has found to be the predominant risk factor for late attempters (*OR* = 2.98; Ayesa‐Arriola et al., 2015). Considering the prevalence and negative outcomes of depression during the post‐acute phase, investigating processes that explain the development of depression with improved insight and identifying therapeutic targets that buffer the insight–depression relationship is warranted (Coentre et al., 2017).

### 1.3 The mediating role of social rank

Researchers have tried to explain the insight–depression relationship, with tentative evidence suggesting a causal relationship whereby insight leads to depression (Hwang et al., 2009). Birchwood et al. (2005) proposed that depression following psychosis may be explained by a psychological response to gaining insight into the illness and its consequences, rather than a symptomatic dimension of the illness itself.

Social rank theory (Gilbert, 1992, 2000, 2020) offers an evolutionary‐based framework to understand the link between insight and depression in the post‐acute phase. According to social rank theory, gaining insight into a highly stigmatised and possibly chronic illness (Kinson et al., 2018) may lead to appraisals involving a loss of social rank, entrapment and defeat, leading to depression. According to Gilbert's evolution‐based model (2014, 2020), humans are motivated to gain social resources (e.g. acceptance and status in the group) and, most importantly, avoid social rejection and exclusion. He posits that a loss in social rank can elicit involuntary low mood and submissive behaviours (e.g. withdrawal and self‐criticism) that are non‐threatening to others and aim to protect the individual from further loss and rejection. However, when an individual feels entrapped and defeated by the situation, these automatic processes do not ‘switch off’ and can progress into depression. In their prospective study, Birchwood et al. (2000) demonstrated that depression following first episode psychosis is predicted by perceived loss of social rank, shame and entrapment but only in those with greater insight. Path modelling has also shown that greater insight into psychosis leads to hopelessness, depression and self‐stigma (Schrank et al., 2014). If gaining insight leads to negative outcomes such as loss in social role, hopelessness, depression, and self‐stigma, it is important to explore modifiable therapeutic targets that mitigate these negative effects.

### 1.4 The moderating role of self‐compassion and mindfulness

Following first‐episode psychosis, self‐compassion appears to be an important process in promoting recovery and growth (Waite et al., 2015). Self‐compassion is the ability to treat oneself with understanding and kindness during hardship (Gilbert, 2009; Neff, 2003). Moving from the competitive mentality involved in social rank to a caring mentality of compassion towards the self (Gilbert, 2014) may facilitate resilience against the stigma of psychosis and buffer the impact of lowered social rank on depression. Those with higher levels of self‐compassion may be more likely to defuse their illness experience from their self‐concept (mindfulness) and recognise that vulnerability is part of the human experience (common humanity; Wong et al., 2019). In unselected university samples, greater self‐compassion (as measured by Neff's, 2003 self‐compassion scale) prospectively predicts lower depression (Raes, 2011) and buffers against depression by reducing rumination (Raes, 2010). Self‐compassion also appears to be a malleable therapeutic target shown to reduce the psychological harm of stigma stress in stigmatised populations (Neff & Germer, 2013; Skinta et al., 2015).

More recently, researchers have suggested that Neff's (2003) self‐compassion scale is more accurately scored as two core components of self‐compassion: compassionate self‐responding (CSR) and uncompassionate self‐responding (UCS, Muris & Otgaar, 2020; Ferrari et al., 2022). Individuals with high CSR are more kind to themselves, recognise that their suffering and imperfection are part of being human, and can take a non‐judgemental, balanced, and accepting stance towards negative thoughts and emotions. By contrast, people with high UCS are highly self‐critical of their mistakes and inadequacies, feel alone in their suffering, and overidentify with negative thoughts and emotions such that they are easily overwhelmed by them. Neff (2020) and Neff et al. (2019) argued that UCS and CSR are simply opposite poles of the self‐compassion continuum (Neff, 2020; Neff et al., 2019). However, others have argued that CSR and UCS are distinct constructs and must not be conflated (Muris & Otgaar, 2020). Indeed, recent studies have demonstrated that when the effects of UCS and CSR on mental health outcomes are examined separately, the detrimental effects of UCS account for far greater variance in mental health outcomes than CSR (e.g. Bicaker & Racine, 2022; Muris et al., 2021). Thus, in addition to examining self‐compassion as a moderator to the association between social rank and depression, following FEP, CSR and UCS may each have an independent moderating effect and should be explored separately.

Mindfulness, or the ability to be aware of and pay attention to what is occurring in the present moment (Brown & Ryan, 2003), may also buffer against depression following psychosis. Mindfulness is a modifiable therapeutic target (Quaglia et al., 2016) that may help individuals to distance from unhelpful ruminative thought processes surrounding social loss and stigma (Brown‐Iannuzzi et al., 2014; Hofmann et al., 2010). Mindfulness‐based interventions have been shown to reduce depressive symptoms in individuals with first episode psychosis and appear well‐tolerated in this population (Jansen et al., 2020; Vignaud et al., 2019). Moreover, mindfulness has shown positive associations with satisfaction with life, self‐efficacy and self‐esteem in young people at ultra‐high risk of psychosis (Alvarez‐Jimenez et al., 2018). However, there have been no published studies that have assessed the role of mindfulness and self‐compassion in the insight–depression relationship. Research in this area may provide preliminary evidence of the relevance of mindfulness and self‐compassion interventions for this population.

### 1.5 The current study

We aimed to investigate whether the adverse relationship between insight and depressive symptoms is explained by social rank in first episode psychosis during the post‐acute recovery phase. Additionally, we examined whether self‐compassion and mindfulness moderated the influence of social rank on depressive symptoms. Symptoms of psychosis have been shown to confound the insight–depression relationship (Murri et al., 2015). Therefore, negative symptoms were controlled for statistically, and we only selected young people in remission to limit the range and influence of positive symptoms.

We hypothesised that (a) consistent with the insight paradox, there will be a significant positive relationship between insight and depressive symptoms, (b) consistent with social rank theory, there will be a significant negative relationship between depressive symptoms and social rank, (c) social rank will mediate the relationship between insight and depressive symptoms, such that greater insight will lead to a loss in social rank, which in turn, leads to greater depressive symptoms, and (d) self‐compassion and mindfulness will moderate the indirect effect of insight on depression via social rank, such that the indirect effect of social rank on depression will be weaker with greater self‐compassion and mindfulness.

## 2 METHOD

### 2.1 Research design

This study utilised secondary data originally collected at the Early Psychosis Prevention and Intervention Centre (EPPIC) in Melbourne as part of the HORYZONS study (Alvarez‐Jimenez et al., 2019; Alvarez‐Jimenez et al., 2021). The HORYZONS study was an 18‐month randomised controlled trial of a moderated online social therapy intervention designed to improve psychosocial outcomes plus treatment as usual, compared to treatment as usual alone. Baseline data used in the current study were collected nearing participants' discharge from EPPIC and prior to randomisation.

### 2.2 Participants

The participants were young people in remission from first episode psychosis and approaching discharge from EPPIC. Relevant inclusion criteria were (a) remission of positive symptoms of psychosis as specified on the Positive and Negative Syndrome Scale (PANSS; Kay et al., 1987); (b) aged between 16 and 27 years; and (c) diagnosis of first episode psychosis or mood disorder with psychotic features, defined by the fourth edition of the Diagnostic and Statistical Manual (DSM‐IV; American Psychiatric Association [APA], 2000). To screen for a manageable level of risk, inclusion criteria also composed of low aggressiveness (i.e. a score of 3 or lower on the PANSS impulse control item) and moderate‐to‐low suicide risk (i.e. a score of 4 or lower on the suicidality subscale of the Brief Psychiatric Rating Scale; Ventura et al., 1993). Relevant exclusion criteria comprised an inability to communicate in English, a DSM‐IV diagnosis of borderline personality disorder or antisocial personality disorder (APA, 2000), and any intellectual impairments or disabilities. For full inclusion and exclusion criteria, refer to Alvarez‐Jimenez et al. (2021).

Of the 170 participants recruited, 25 did not complete the measures of interest. Therefore, the final sample comprised 66 females and 79 males (*N* = 145) aged between 16 and 27 (*M* = 20.81, *SD* = 2.85). Independent sample *t*‐tests and Pearson's chi‐square test of contingencies indicated no significant differences between those who did not complete measures of interest and those who did, in age, sex, duration of untreated psychosis, and visits to emergency and medical services. Duration of untreated psychosis averaged 27.42 weeks (*SD* = 56.53). See Table 1 for further participant demographic and illness characteristics.

**TABLE 1:** Participant characteristics (*n* = 145).

Variable	*n* (%)
Gender	
Female	66 (45.5)
Male	79 (54.5)
Educational status	
Not currently studying	18 (24.3)
Enrolled in upcoming course	11 (32.8)
Part‐time study	37 (31.4)
Full‐time study	10 (11.5)
Employment status	
Paid work only	28 (19.3)
Study only	35 (24.1)
Paid work and study	23 (15.9)
Unemployed	41 (28.3)
Educational attainment in high school	
Year 8	2 (1.4)
Year 9	12 (8.3)
Year 10	31 (21.4)
Year 11	31 (21.4)
Year 12	68 (46.9)
Diagnosis	
Affective psychosis	61 (42.1)
Non‐affective psychosis	84 (57.9)

### 2.3 Measures

The data from the following baseline measures were obtained from the HORYZONS study.

#### 2.3.1 Depression

The Calgary Depression Scale for Schizophrenia (CDSS; Addington et al., 1993) is a 9‐item interview that assesses severity of depressive symptoms, independent of antipsychotic medication side effects and negative symptoms. Items are rated on a 4‐point Likert scale ranging from 0 (*absent*) to 3 (*severe*). Scores are averaged, with higher scores indicating greater levels of depression. The CDSS is commonly used for severity of depression in psychosis and has good psychometric properties (Addington et al., 1993; Lako et al., 2012). In the current sample, internal consistency was *α* = .83.

#### 2.3.2 Self‐compassion

The Self‐compassion Scale Short Form (SCS‐SF; Raes et al., 2011) is a 12‐item self‐report measure that assesses the ability to be kind and accept oneself when experiencing suffering (Neff, 2003). Items are rated using a 5‐point Likert‐type scale ranging from 1 (*almost never*) to 5 (*almost always*). The SCS‐SF has demonstrated a near‐perfect correlation with the long form, good construct validity and excellent internal consistency (Hayes et al., 2016; Raes, 2011).

Recent research suggests that the positively worded items of the SCS‐SF reflect a compassionate style of self‐responding, composed of self‐kindness, common humanity and mindfulness. In contrast, the negatively worded items of the self‐judgement, isolation and overidentification subscales operationalise a different construct, reflecting an uncompassionate style of self‐responding rather than merely an absence of self‐compassion (Muris et al., 2019; Muris & Otgaar, 2020; Muris & Petrocchi, 2017). We therefore follow recent recommendations by Ferrari et al. (2022) and report results using the total score SCS‐SF in addition to examining whether the two subscales CSR and UCS have different effects. The internal consistency of the CSR and UCS in the current sample was acceptable (*α* = .76) and very good (*α* = .86), respectively.

#### 2.3.3 Mindfulness

The Mindfulness Attention Awareness Scale (MAAS; Brown & Ryan, 2003) is a 15‐item self‐report measure assessing the frequency of awareness and attention to what is happening in the present moment. Items are rated using a 6‐point Likert scale and range from 1 (*almost always*) to 6 (*almost never*). The MAAS has shown excellent test–retest reliability and strong internal consistency (Brown & Ryan, 2003) and has good psychometric properties in first‐episode psychosis samples (González‐Blanch et al., 2022). The internal consistecy of the measure in the current sample was excellent (*α* = .98).

#### 2.3.4 Insight

An abbreviated version of the Scale to Assess Unawareness of Mental Disorder (SUMD; Amador et al., 1993) was used to assess level of insight into psychosis. The SUMD is the most used measure of insight in psychosis research (Elowe & Conus, 2017), showing good reliability and validity (Amador et al., 1993). It is a clinician‐rated scale that differentiates between awareness of current symptoms and awareness of symptoms from their most recent episode (retrospective insight). As this study followed participants in the post‐acute phase, only retrospective insight was assessed. The original SUMD assessed general insight (3 items) and insight into 17 symptoms. In our current study, only insight into four symptoms was assessed: hallucinations, delusions, flat or blunted affect, and asociality. Insight into these symptoms was assessed as they are the most frequently reported symptoms in young people with early psychosis. Awareness of symptoms and the attribution of symptoms were assessed for each symptom and formed two subscales—the awareness of symptoms and the attribution of symptoms subscales. Items are rated by clinicians using a 5‐point scale ranging from 1 (*completely aware*) to 5 (*completely unaware*). Higher scores indicate poorer insight. In the current sample, the internal consistency for awareness of symptoms subscale was acceptable (*α* = .64) and was used as the measure of insight. The attribution of symptoms to psychosis subscale was not used due to poor internal consistency (*α* = .37).

#### 2.3.5 Social rank

The Social Comparison Scale (SOCPS; Allan & Gilbert, 1995) is an 11‐item self‐report questionnaire derived from social rank theory (Gilbert, 1992, 2000) that measures perceived social rank relative to others. Items are rated on a 10‐point bipolar scale based on how respondents perceive themselves relative to others (e.g. inferior–superior). The SOCPS has been utilised in early psychosis research (e.g. Allison et al., 2013), demonstrating good internal consistency both previously (Allan & Gilbert, 1995) and in the current sample (α = .91).

#### 2.3.6 Negative symptoms

The PANSS (Kay et al., 1987) is a 30‐item interview used to measure the severity of positive and negative symptoms of psychosis. The negative symptoms subscale comprises seven items. Items are rated by clinicians on a 7‐point Likert scale ranging from 1 (*absent*) to 7 (*extreme*). The PANSS has demonstrated excellent validity and reliability (Kay et al., 1987). The negative symptoms subscale showed adequate internal consistency (*α* = .65) and inter‐rater reliability (.89).

### 2.4 Procedure

The Melbourne Health Human Research Ethics Committee (2013.146) provided ethics approval for the primary study from which the data were obtained and provided access to the de‐identified dataset for the current study. The current study was additionally approved by the Australian Catholic University Human Research Ethics Committee (2021‐167N). The recruitment phase ran across 28 months from September 2013 to December 2015. Lists of young people nearing discharge from EPPIC were provided to the HORYZONS study coordinator and assessed for initial eligibility in consultation with the young person's treating clinician and case managers. Individuals considered initially eligible were approached to obtain informed consent and undergo a screening assessment to finalise eligibility. The participants who met eligibility criteria completed baseline assessments and were subsequently randomised. Refer to Alvarez‐Jimenez et al. (2021) for details of recruitment and participant flow prior to randomisation as well as mechanisms to assess inter‐rater reliability of the CDSS and PANSS.

### 2.5 Data analysis

The study is a cross‐sectional correlational study. Data analysis was conducted using SPSS v26. Firstly, missing values analyses were undertaken and assumptions for bivariate correlation analysis and linear regression were tested. Secondly, descriptive statistics and internal consistency of questionnaires were computed. Thirdly, correlation coefficients were calculated to assess the relationship between variables; notably, between insight and depression (H1) and depression and social rank (H2).

Finally, the PROCESS macro (Hayes, 2018) was used for all conditional process analyses. All models had depression as the dependent variable, and the PANNS negative symptoms score as a covariate. Predictor variables were centred and significant interactions were probed using simple slopes analyses at ±1 standard deviation from the mean to test for moderating effects (Aiken & West, 1991).

## 3 RESULTS

### 3.1 Preliminary analysis

A missing value analysis revealed minimal missing data (< 5%) of item responses within scales, and Little's MCAR tests were nonsignificant, indicating the data were missing completely at random. Missingness was addressed by replacing missing items with the mean score of available items within the scale or subscale (Tabachnick & Fidell, 2013). Assumptions of normality, linearity, and homoscedasticity for bivariate correlations analysis using Pearson's r were violated. Correlations between model variables were therefore tested using Spearman's Rho. Assumption testing for linear regression were also undertaken. Three univariate outliers above 3.29 standard deviations of the mean were detected for the awareness of psychosis subscale of the SUMD, four for the negative symptoms scale of the PANSS, seven for CDSS, three for SCSS‐SF and one for SOCPS. These univariate outliers were adjusted to 3.29 standard deviations above the mean (Tabachnick & Fidell, 2013). Four cases above the critical *χ* ^2^ value for *df* = 7 (at *α* = .001) of 24.32 were detected through inspection of Mahalanobis distances. However, Cook's distances were less than one, indicating these outliers did not require addressing (Pallant, 2010). Violated assumptions of multivariate normality, linearity, and heteroscedasticity were addressed using robust standard errors HC3 (Long & Ervin, 2000) (Hayes, 2018). Correlations between variables and descriptive statistics are presented in Table 2. Of the total sample, almost one in four (24.2%) participants presented with clinically significant depressive symptoms using the cut‐off of 6 or more on the CDSS (Addington et al., 1993; Phahladira et al., 2021; Sönmez et al., 2016). Most participants (77%) were unaware of their illness symptoms, as indicated by a score of 1 on the SUMD (Amador et al., 1993).

**TABLE 2:** Means, standard deviations and correlations among variables (*n* = 145).

Variable	*M*	*SD*	1	2	3	4	5	6	7	8
1. Awareness of psychosis	3.18	2.41	‐							
2. Depression	3.20	3.40	.048	‐						
3. Social rank	59.50	17.11	−.062	−.390 **	‐					
4. Negative symptoms	11.11	3.30	.541 **	.213 *	−.217 *	‐				
5. Mindfulness	3.73	0.99	−.061	−.365 **	.426 **	−.120	‐			
6. Self‐compassion total score	34.66	8.04	−.163	−.430 **	.548 **	−.197 *	.490 **	‐		
7. Compassionate self‐responding	19.08	4.60	−.125	−.263 **	.390 **	−.090	.281 **	.691 **	‐	
8. Uncompassionate self‐responding	20.37	5.61	.105	.442 **	−.456 **	.167 *	−.461 **	−.851 **	−.262 **	‐

* *p* < .05.

** *p* < .001.

### 3.2 Hypothesis testing

#### 3.2.1 Correlations between primary variables

Contrary to the insight paradox (H1), there was no significant relationship between insight and depressive symptoms, *r*_*s*_(167) = .05, *p* = .56. Consistent with the social rank theory (H2), there was a significant negative relationship between social rank and depressive symptoms, with a medium effect size, *r*_*s*_(145) = −.40, *p* = <.001). In relation to the SCS, across all correlations UCS tended to have a stronger and significant relationship with awareness of psychosis, depression, social rank, negative symptoms and mindfulness compared to the CSR subscore. Given that there was no significant association between insight and depression (i.e. no total effect of insight on depression in this sample), mediation was not possible.

#### 3.2.2 Testing for moderated effect

Despite no evidence of a mediating effect, it is still possible that self‐compassion and mindfulness could moderate the relationship between social rank and depression (Hayes, 2018). Results of direct effects and interactions of the hypothesised moderated relationships (H4) are shown in Table 3. Both self‐compassion and mindfulness negatively predicted depression even after accounting for negative symptoms. However, only a significant interaction between self‐compassion and social rank emerged, Δ*R* ^2^ = .03, *F* (1, 138) = 5.11, *p* = .03. The interaction between social rank and mindfulness on depression was non‐significant, Δ*R* ^2^ = .003, *F* (1, 138) = .41, *p* = .52.

**TABLE 3:** Results of moderation analyses for factors predicting depression severity (*n* = 145).

	*b*	SE (HC3)	*t*	*p*	95% CI
Main analysis					
Social rank	−0.04	0.02	−1.74	.09	−0.74, 0.01
Self‐compassion (SCS)	−0.12	0.05	−2.36	.02	−0.21, −0.02
Mindfulness	−0.62	0.29	−2.10	.04	−1.20, −0.04
Negative symptoms	0.07	0.08	0.92	.36	−0.08, 0.23
Social rank × SCS	0.01	0.002	2.26	.03	−0.21, −0.02
Social rank × mindfulness	−0.01	0.02	−0.64	.52	−0.05, 0.03
Exploratory analysis					
Social rank	−0.05	0.02	−2.09	.04	−0.09, −0.002
UCS	0.22	0.06	3.46	.001	0.09, 0.34
CSR	−0.06	0.06	−0.95	.34	−0.18, 0.06
Negative symptoms	0.09	0.08	1.13	.26	−0.07, 0.24
Social rank × UCS	−0.01	0.003	−2.24	.03	−0.01, −0.001
Social rank × CSR	0.00	0.00	−0.04	.97	−0.01, 0.01

*Note*: UCS = uncompassionate self‐responding, CSR = compassionate self‐responding. The main analysis examined whether self‐compassion and mindfulness moderated the relationship between social rank and depression. The exploratory analysis examined whether UCS and CSR moderated the relationship between social rank and depression. All predictor variables were centred, and HC3 standard errors were used.

**FIGURE 1:**
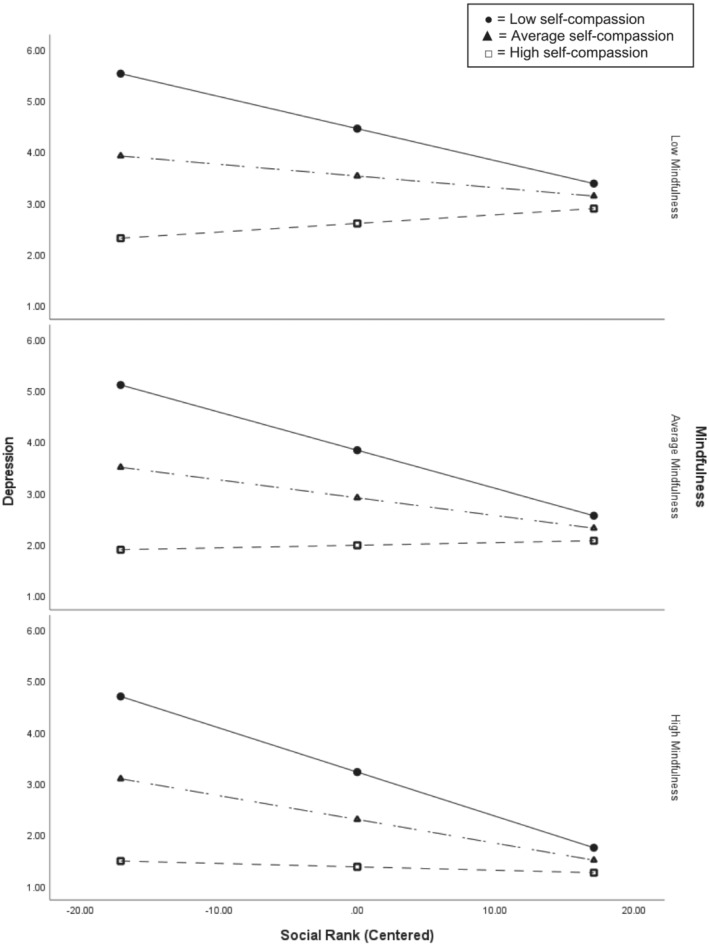
Simple slopes demonstrating that the relationship between social rank and depression is moderated by self‐compassion.

The significant interaction between social rank and self‐compassion on depression was probed using simple slopes analyses at ±1 standard deviation from the mean (Aiken & West, 1991) and revealed that the relationship between social rank and depression was stronger for lower levels of self‐compassion, and that at high levels of self‐compassion, individuals reported low depression regardless of social rank (see Figure 1). The final model explained 33% of the variance in depression.

### 3.3 Exploratory analyses

**FIGURE 2:**
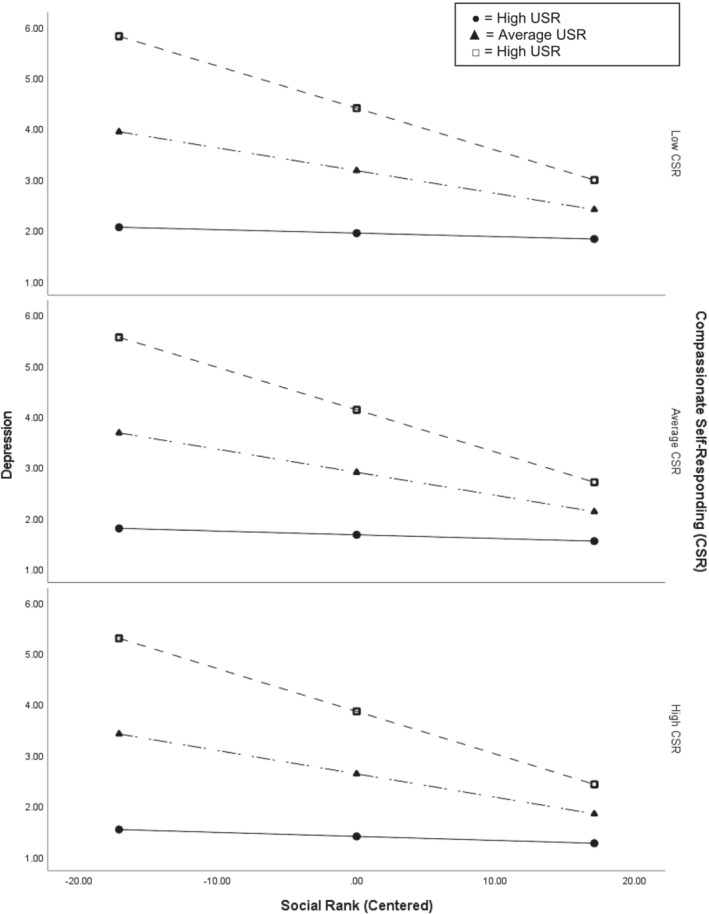
Simple slopes demonstrating that the relationship between social rank and depression is moderated by uncompassionate self‐responding.

To explore which aspects of self‐compassion moderated the relationship between social rank and depression, we conducted moderation analyses with CSR and UCS as moderators to the relationship between social rank and depression. The results showed that only UCS significantly moderated the relationship between social rank and depression. Simple slopes analysis showed that the negative association between social rank and depression was only significant for high levels of UCS (see Figure 2).

## 4 DISCUSSION

Situated within social rank theory (Gilbert, 1992, 2000), this study examined the relationship between insight and depression during the post‐acute recovery phase of first episode psychosis, and the role of social rank, self‐compassion, and mindfulness in this relationship. Unexpectedly, insight was not related to depression in this sample (H1). Social rank was negatively related to depression (H2), but no mediation analysis was conducted as there was no insight–depression relationship (H3). Whilst there was no moderated mediation effect (H4), we found that UCS moderated the pathway between social rank and depression.

Our findings on the insight–depression link are inconsistent with previous research examining psychosis in the post‐acute phase (e.g. Murri et al., 2015; Phahladira et al., 2021). This may be because we excluded people with high levels of suicidality in our study. It is possible that this exclusion may have dampened the magnitude of the insight–depression relationship. Furthermore, a meta‐analysis has shown that the insight–depression link has a small effect size (*r* = .14; Murri et al., 2015), and only increased from 13% to 25% of explained variance after controlling for confounding variables. Thus, whilst the range of positive symptoms was restricted in the current study, variables not measured may have affected the strength and significance of the relationship observed (e.g. economic status, Murri et al., 2016).

It is important to note that other studies have also failed to demonstrate a significant relationship between insight and depression at 2 years following first psychosis presentation (e.g. Crumlish et al., 2005). Interestingly, in a recent RCT on the effects of early intervention for first episode psychosis on insight, DeTore et al. (2022) found the presence of the insight–depression paradox in the whole sample at baseline. However, at 6 months follow‐up, there was no association between insight and depression among individuals who participated in the early intervention, indicating that early intervention might reduce the insight–depression paradox. Whilst the present study used the current gold standard measure of insight, administered by clinical interview, challenges in accurately measuring the subjective experience of an individual experiencing a powerful impairment in functioning must be acknowledged. The lack of association between insight and depression in our current study might likewise have been due to the involvement of participants in an early intervention programme.

Previous findings (Crumlish et al., 2005; DeTore et al., 2022), together with the findings of the current study, raise questions regarding the importance of insight in depression during this phase of illness. It is possible that over time and with early intervention, the negative effects of insight on depression begin to wane and insight no longer affects depression. Other factors such as perceived social rank, self‐esteem, and stigma may take over from insight as people recovering from first episode psychosis try to re‐engage in social, functional, and vocational life activities (Cogan et al., 2019; Waite et al., 2015; Windell & Norman, 2013). Together with the current study, the conflicting results in the literature suggest that the insight–depression relationship may be more complex and time‐sensitive than first considered, and other processes could play a more important role in contributing to depression during recovery of first episode psychosis.

Like previous study samples (e.g. Phahladira et al., 2021), depression in the post‐acute stage of first episode psychosis was common for the present sample, with almost one in four participants presenting with clinically significant symptoms. Whilst social rank did not mediate the relationship between insight and depression, it was directly related to depression. These results support social rank theory (Gilbert, 1992, 2000) and align with previous studies investigating the association between social rank and depression across different presentations (Wetherall et al., 2019). Our results suggest that understanding an individual's perceived social rank and changes because of experiencing psychosis may be an important process in depression aetiology and maintenance, particularly for those who experience an event where a loss in social standing ensues. From this perspective, interventions to assist these young people improve their perception of self in relation to others and buffer the negative effect of low social rank are needed to prevent or reduce depression. Our results indicate that this may be achieved by directly targeting perceived social rank and/or through decreasing UCS.

Higher levels of self‐compassion may buffer the threat defence system activated by perceived low social rank (Gilbert, 1992, 2000, 2020) as self‐compassion is not dependant on self‐evaluation or upward social comparison, but on the notion that everyone is flawed and deserves compassion and understanding (Neff & Vonk, 2009). Therefore, even with a loss in social rank, depression does not significantly increase because self‐compassion reduces the salience of social rank. Our findings support the rationale for implementing compassion‐focused interventions during the post‐acute recovery phase of psychosis. Indeed, compassion‐focused interventions have demonstrated significant improvements in self‐compassion, social rank, depression, and shame in psychosis populations (Braehler et al., 2013; Laithwaite et al., 2009). Alternatively, the negative relationship between social rank and depression suggests that interventions directly targeting perceptions of low social rank may also lead to a reduction in depression. For example, cognitive behaviour therapy could challenge beliefs related to low social rank (e.g. I am inferior relative to others, Gumley et al., 2010).

Our exploratory analyses showed that UCS (but not CSR) had the strongest direct effects on depression and significantly moderated the association between social rank and depression. The correlation analyses showed a stronger and significant relationship between UCS and all other outcomes compared to CSR. The simple slopes analysis showed that the relationship between social rank and depression was no longer significant at low levels of UCS. These exploratory analyses suggest differential effects of specific aspects of self‐compassion. A growing body of research suggests that much of the strength of the SCS measure in predicting psychological and mental health outcomes is due to the UCS items, which are more closely related to psychopathology than the CSR items (Muris et al., 2019, 2016; Muris & Otgaar, 2020). The individual scale items forming the UCS of the SCS target self‐response styles that are belittling, self‐critical, induce loneliness, and encourage a fixation or over‐identification with thoughts and feelings, particularly those that are unhelpful. Based on past research and the current findings, it is unclear whether these negative items are measuring the presence of UCS (which is likely to present in a similar fashion to being very self‐critical) or are also measuring a general lack of self‐compassion. Whilst the present study does not seek to resolve this broader debate about the UCS subscale, the present findings do suggest that for individual's recovering from psychosis, identifying UCS trends may be helpful to understand the relationship between insight and depression.

The role of UCS in moderating the link between lower perceived social rank and depression symptoms aligns with an emerging body of research suggesting perceived social rank is an important consideration in populations who experience psychosis. Heriot‐Maitland et al. (2019) found that the positive symptoms of psychosis, specifically auditory hallucinations, are often experienced as a persecutory voice and may reflect the activation of the threat‐based motivational system. As a result, compassion‐focused therapy is an effective way to understand the function and effect of these symptoms, and cultivate a more compassionate self‐identity to create internal patterns of security and compassion (Heriot‐Maitland, 2020). The present study suggests that such benefits may also be worthwhile for those who are in remission and recovering from positive psychosis symptoms, as were the present sample.

The current results also further support the argument to use the UCS and CSR as an alternative to using the total score in isolation (Ferrari et al., 2022; Muris et al., 2019, 2016; Muris & Otgaar, 2020). The pattern of results in the current study suggests that the presence of an uncompassionate style of relating to one's self, or absence of self‐compassion, is a more powerful predictor of depression than the presence of a tendency to be kind to one's self. Such understandings could inform future studies regarding psychosis symptom progression and the development of targeted psychological interventions. Indeed, it may be the case that self‐compassion interventions which specifically target and are effective in reducing self‐critical ways of self‐responding may be of benefit for this population over interventions which specifically target an increase in CSR (Ferrari et al., 2019). In support of social rank theory (Gilbert, 2020), these results suggest that with a loss in social rank, deactivating the self‐critical, competitive mentality is important for buffering progression to depression. Future research could further investigate the role of entrapment and defeat in this process.

Whilst our results indicate that one facet of mindfulness (awareness and attention to the present moment) did not moderate the effects of social rank on depression, it is possible that other facets of mindfulness (e.g. non‐judgement and acceptance) could have buffered this relationship. Indeed, other facets of mindfulness have shown to buffer perceived discrimination on depression in community samples (Brown‐Iannuzzi et al., 2014). Aside from *how* one pays attention, our results may have been explained by *what* our sample was paying attention to, with evidence suggesting that attention to suffering in depression (e.g. rumination and worry) is linked to worse depression (Desrosiers et al., 2014). Nonetheless, in our sample, the awareness facet of mindfulness was still negatively associated with depression and therefore could still be a valuable therapeutic target for reducing depression following the first episode psychosis (Reich et al., 2021). Our results suggest that bringing mindful awareness to internal experiences may be an important step to mitigating depression; however, uncompassionate responding (e.g. self‐criticism) appears to be the key ingredient influencing the effects of low social rank on depression. Future studies could conduct experimental manipulations of the separate components of self‐compassion and mindfulness (e.g. Dreisoerner et al., 2020) to test these assertions and assess subsequent changes in social rank and depression during the course and recovery from first episode psychosis.

### 4.1 Limitations

The current findings should be considered in the context of several limitations. Firstly, the cross‐sectional design precludes any causal inferences between social rank and depression. Reverse causality may be possible. However, the strong theoretical and empirical underpinning of the current study make this less likely. Nonetheless, future studies could further elucidate this relationship by investigating the temporal order and association between social rank and depression over the course of first episode psychosis and include additional relevant variables such as entrapment. Secondly, there were limitations inherent to using a pre‐existing data set. Controlling for potential confounders previously shown to increase the magnitude of the insight–depression relationship may have provided a more accurate observation of this relationship. Additionally, only young people with low aggressiveness and positive symptoms, and low‐to‐moderate suicidality were eligible to participate, reducing the generalisability of the findings.

Given that our study focused on patients who were in remission from the positive symptoms of psychosis, we did not investigate the effects of social rank and power relationships to voice hearing in psychosis, to which social rank theory has been applied (Birchwood et al., 2002, 2006; Birchwood, Meaden, et al., 2000). Future research examining these factors and how they may affect depression following FEP is recommended.

Finally, limitations in the measures should be noted. The use of an abbreviated insight measure may have limited the ability to assess subtle variations in insight, and the MAAS only allowed us to investigate one facet of mindfulness (Baer et al., 2006). Future studies should investigate the role of other components of mindfulness in social rank and depression in psychosis. In addition, the psychometric properties of the self‐compassion scale have not be examined for the current population. Further research such as a confirmatory factor analysis on the self‐compassion scale is required. In addition, the current study found that UCS moderated the relationship between social rank and depression symptoms, but not CSR. Although Neff (2003) proposes UCS and CSR operate in tandem with increases in CSR automatically resulting in decreases in UCS and vice versa, more recent research suggests this may not always be the case (Ferrari et al., 2022; Muris et al., 2019; Muris & Otgaar, 2020; Muris & Petrocchi, 2017). Further intervention research could examine whether psychological interventions seeking to increase self‐compassion (CSR) provide benefit for those in recovery from first‐episode psychosis.

### 4.2 Conclusion

In summary, the role of insight in depression appears unclear, with the current results countering those in previous studies. Our results suggest that insight during the post‐acute phase is less important in depression than previously considered. Nonetheless, this study highlights the role of social rank in depression in first‐episode populations, providing further support for social rank theory and underscoring the detrimental effects of UCS. How mindfulness influences depression is less clear. UCS social rank and possibly mindfulness may be valuable treatment targets to reduce depression and improve recovery for these young people. Further intervention beyond standard early psychosis interventions may be needed, considering the prevalence of depressive symptoms during this phase.

## CONFLICT OF INTEREST STATEMENT

We have no conflicts of interest to disclose.
